# Comprehensive metabolomic characterization of patients with myasthenia gravis

**DOI:** 10.3389/fimmu.2026.1906581

**Published:** 2026-09-29

**Authors:** Hui Deng, Yuexuan Wang, Xiaowen Yang, Jing Qin, Jing Liu, Tianlong Liu

**Affiliations:** 1 Department of Neurology, Affiliated Hospital of Inner Mongolia Medical University, Hohhot, China; 2 College of Pharmacy, Inner Mongolia Medical University, Hohhot, China; 3 Department of Pharmacy, Affiliated Hospital of Inner Mongolia Medical University, Hohhot, China; 4 Department of Endocrinology, Affiliated Hospital of Inner Mongolia Medical University, Hohhot, China; 5 Key Laboratory of Clinical and Basic Research on Cardiovascular Diseases, Basic Research Team of Cardiovascular Diseases, Affiliated Hospital of Inner Mongolia Medical University, Hohhot, China; 6 Inner Mongolia Autonomous Region Institute of Cardiovascular Diseases, Hohhot, China

**Keywords:** biomarkers, diagnostic value, metabolic alteration, myasthenia gravis, plasma metabolomic characterization

## Abstract

**Background:**

Myasthenia gravis (MG) is mediated by abnormal antibody generation and is characterized by muscle weakness and pathological fatigue. However, the accuracy of anti-acetylcholine receptor (AChR) antibody testing for the diagnosis of MG needs improvement.

**Objective:**

This study was aimed to systematically characterize the plasma metabolome of patients with MG and identify novel biomarkers with significant diagnostic value.

**Methods:**

A cohort of 130 subjects was enrolled and divided into discovery and validation sets, and their plasma metabolomic profiles were determined by liquid chromatography–tandem mass spectrometry. Metabolomic variables were analyzed along with clinical information to identify relevant diagnostic biomarkers. The diagnostic efficacy of the screened metabolites and biomarker panel was assessed via receiver operating characteristic (ROC) curve analysis.

**Results:**

In positive and negative ion modes, plasma metabolomic profiling revealed 326 and 398 differentially abundant metabolites, respectively, in the discovery and validation sets. The metabolomic profile distinguished patients with MG from healthy controls across the two cohorts evaluated in this study. Furthermore, 890 metabolites were detected in both the discovery and validation sets. Metabolite set enrichment analysis of these 890 metabolites revealed that steroid hormone biosynthesis pathway was significantly enriched, implying that altered metabolism may serve as a potential pathological hallmark of MG. On the basis of the differences between groups, compound level, the value of the variable importance in projection, the area under the ROC curve (AUC) and precision–recall curve analyses, 3 differentially abundant metabolites, namely, LysoPE(P-18:1/0:0), PE(O-18:1/0:0) and PE(P-16:0/0:0), were selected as a biomarker panel to distinguish between patients with MG and healthy controls. ROC curve (AUC) analysis revealed that the biomarker panel and anti-AChR antibody testing had equivalent MG diagnostic ability (AUC: 0.883 vs. 0.825; *P* = 0.058 in the discovery set; AUC: 0.843 vs. 0.722; *P* = 0.121 in the validation set); moreover, the combination of the biomarker panel and anti-AChR antibody testing significantly improved the clinical diagnosis of MG in the discovery and validation sets compared with anti-AChR antibody testing alone.

**Conclusion:**

Our screened biomarker panel discriminated patients with established MG from healthy healthy controls, however, further evaluation in independent cohorts including patients with suspected MG and other relevant autoimmune and neuromuscular junction disease healthy controls is warranted.

## Introduction

Myasthenia gravis (MG) is a rare, chronic, neuroimmunological disease caused by autoantibodies that affect neuromuscular junctions and result in muscle weakness and fatigability (1). MG is caused by autoantibodies that target acetylcholine receptor (AChR) and muscle-specific kinase (MuSK) proteins that damage neuromuscular transmission (2, 3). Therefore, the peripheral blood of some patients with MG test positive for anti-AChR and anti-MuSK antibodies, whereas patients with MG who are negative for these antibodies are defined as having seronegative MG (4). An epidemiological investigation revealed that more than 700,000 individuals have been diagnosed with MG worldwide, with the prevalence ranging from 50–200 per 1,000,000 (5). The main clinical characteristics and complications of MG are diverse and change with increasing age (6, 7). Generally, 50 years is suggested as the cutoff age to distinguish between early-onset and late-onset disease (8). Early-onset MG is generally characterized by thymic hyperplasia, whereas thymic atrophy is a hallmark of late-onset MG (9, 10). Despite advances in acetylcholinesterase inhibitors and broad immunosuppressants, e.g., glucocorticosteroids and nonsteroidal immunosuppressive therapies, avoiding long-term adverse effects and achieving significant clinical symptom improvement remain challenging (11–13).

Another challenge for patients with MG is obtaining an accurate diagnosis. Although more than half of patients with generalized and ocular MG tested positive for anti-AChR antibodies, plasma anti-AChR antibody levels were not associated with MG progression, severity or treatment response (14, 15). Recently, researchers have begun to focus on additional biomarkers that reflect MG progression and severity, such as circulating microRNAs and metabolites (16–18). Previous studies have proposed that alterations in components of amino acid metabolism, the tricarboxylic acid (TCA) cycle, and glycerophospholipid metabolism could be potential biomarkers for the diagnosis of MG (15). In addition, certain plasma proteins, such as interleukin (IL)-10, the IL-12 family of cytokines, IL-12p40, IL-20, IL-22 and IL-26, are potential biomarkers for MG (19). In our study, a nontargeted metabolomics approach was applied to identify and subsequently validate the plasma metabolic characteristics of patients with MG and assess the diagnostic performance of the identified biomarkers to distinguish patients with and without MG, providing a theoretical basis for further interpreting the pathological mechanism underlying MG.

## Materials and methods

This study was performed in accordance with the Declaration of Helsinki and was approved by the Ethics Committee of the Affiliated Hospital of Inner Mongolia Medical University (No. 2016-732). All the participants provided written informed consent.

### Study participants and blood samples

This study included consecutive patients with MG who were admitted to the neurology outpatient and inpatient departments of the Affiliated Hospital of Inner Mongolia Medical University from April 2023 to October 2024. Detailed records of these patients were collected. The inclusion criteria were as follows (1): met the Chinese expert consensus on the diagnosis and treatment of MG, which includes fluctuating muscle weakness and fatigue, a positive neostigmine test, and a decrease in the amplitude of low-frequency, repetitive nerve stimulation exceeding 10% (2); experienced benefit from cholinesterase inhibitors (3); underwent a radioimmunoprecipitation assay (most patients with MG are positive for plasma anti-AChR antibodies; only a minority of patients with MG are positive for anti-MuSK and anti-LRP4 antibodies) (4); underwent a CT examination for thymoma; and (5) did not have a history of treatment with steroids, immunosuppressive agents, or plasma exchange within the past 3 months. The exclusion criteria were as follows (1): severe heart, liver, kidney, or lung insufficiency (2); other severe diseases, e.g., malignant tumors, cerebral infarction and hemorrhage, asthma, pulmonary hypertension, or metabolic diseases other than diabetes, and a lack of clinical data; and (3) the presence of other autoimmune diseases or the use of drugs that suppress immune function. Ultimately, 65 MG patients were enrolled in the study; 20 MG patients were randomly selected to form the discovery set, and the remaining 45 patients formed the validation set. Age- and sex-matched healthy controls were recruited at the Health Examination Center of the Affiliated Hospital of Inner Mongolia Medical University; none of the healthy controls had any history of autoimmune disease or had been treated with steroids or immunosuppressants during the past year, and all of them exhibited normal neurological and systemic medical findings. The quantitative MG score (QMGS) evaluation, Myasthenia Gravis Foundation of America (MGFA) class and MG Activities of Daily Living (MG-ADL) score was performed by a physician with experience in neuromuscular diseases.

Peripheral venous blood samples from the patients with MG and healthy controls were stored in K2 EDTA Vacutainer tubes and immediately cooled to 4 °C. The samples were then centrifuged at 3000 × g for 10 min at 4 °C within 2 h. Supernatants (plasma) were separated, transferred to new vials, immediately frozen and stored at −80 °C until sample preparation.

### Nontargeted metabolomics

#### Sample preparation

The samples stored at −80 °C were thawed at room temperature. One hundred microliters of the sample was added to a 1.5 mL Eppendorf tube with L-2-chlorophenylalanine (0.06 mg/mL) dissolved in acetonitrile and methanol (V:V=2:1) as an internal standard, and the tube was vortexed for 1 min. Subsequently, ultrasonic extraction was performed in an ice-water bath for 10 min, followed by overnight incubation at −40 °C. The extract was then centrifuged at 4 °C (12,000 rpm) for 10 min. Next, 150 µL of the supernatant was filtered through 0.22 µm microfilters and transferred to Liquid chromatography vials. The vials were stored at −80 °C until LC–MS analysis. QC samples were prepared by mixing aliquots of all the samples to generate a pooled sample.

#### LC–MS/MS analysis

Metabolomic data analysis was performed by Shanghai Luming Biological Technology Co., Ltd. (Shanghai, China). An ACQUITY UPLC I-Class PLUS (Waters Corporation, Milford, USA) coupled to a Q Exactive mass spectrometer equipped with a heated electrospray ionization (ESI) source (Thermo Fisher Scientific, Waltham, MA, USA) was used to analyze the metabolic profile in both positive and negative ion modes. An ACQUITY UPLC HSS T3 column (100 mm×2.1 mm, 1.8 um) was used for separation. Binary gradient elution was performed with solvents (A) water containing 0.1% v/v formic acid and (B) acetonitrile as follows: 0.01 min, 5% B; 2 min, 5% B; 4 min, 30% B; 8 min, 50% B; 10 min, 80% B; 14 min, 100% B; 15 min, 100% B; 15.1 min, 5% B; and 16 min, 5% B. The flow rate was 0.35 mL/min, and the column temperature was 45 °C. All the samples were maintained at 10 °C during analysis. The injection volume was 3 μL.

The mass spectrometry detection range was m/z 70–1050 in both positive and negative ion modes. The resolution was set at 70,000 for the full MS spectra and 17,500 for HCD MS/MS. The collision energy was set to 10, 20 and 40 eV. The mass spectrometer was operated with the following parameters: spray voltage, 3800 V (+) and 3200 V (−); sheath gas flow rate, 35 arbitrary units; auxiliary gas flow rate, 8 arbitrary units; capillary temperature, 320 °C; aux gas heater temperature, 350 °C; and S-lens RF level, 50.

#### Data preprocessing

The raw LC–MS data were processed using Progenesis QI V2.3 software (Nonlinear, Dynamics, Newcastle, UK) by performing baseline filtering, peak identification, peak integration, retention time correction, peak alignment, and peak normalization. The main parameters applied were a precursor tolerance of 5 ppm, a product tolerance of 10 ppm, and a product ion threshold of 5%. Compounds were identified based on the precise mass-to-charge ratio (m/z), secondary fragments, and isotopic distribution using The Human Metabolome Database (HMDB), LIPID MAPS (V2.3), METLIN, and in-house databases.

#### Qualitative and quantitative analyses

The obtained data were subjected to relative standard deviation (RSD) filtering, missing value treatment, zero value replacement, score filtering, log_2_ transformation and data merging. The ion peak from quality control samples with RSD values greater than 30% were removed. Ion peaks for which the proportion of missing values (zero values) within a group exceeded 50% were removed; the remaining zero values were replaced with half the minimum ion intensity value across all samples. The data were subsequently subjected to log_2_ transformation. A score was assigned to each compound based on the qualitative results, and the compounds identified through qualitative analysis were screened. The compound scoring was defined as following: The maximum score is 80 marks, exact molecular weight matching in the first mass spectrometry (20 marks), fragment matching in the secondary mass spectrum (20 marks), isotopic distribution matching (20 marks), and retention time matching (20 marks). It is generally accepted that the higher the score, the more accurate the identification.The screening criterion was 36 points out of a maximum of 80 points; compounds with a score less than 36 points were considered to have inaccurate qualitative data and were removed from further analysis. The compound level was defined as following (1): Level 1:RT: ± 0.3 min (18s) and Fragmentation Score ≥ 45; (2) Level 2: RT: ± 0.3 min (18 s) and 0 ≤ Fragmentation Score < 45; (3) Level 3: Fragmentation Score ≥ 45; (4) Level 4: Fragmentation Score < 45. In our study, the metabolites with level 1 were selected as potential biomarkers and further validation.

### Statistical analysis

Data for continuous variables are displayed as the means ± standard deviations (SDs). Statistical comparisons of percentages were performed using χ^2^ analysis. The statistical analysis workflow of the nontargeted metabolomics data is shown in **Supplementary Figure S1**. Principal component analysis (PCA) and Bayesian factor analysis (with normalized factor score plots) were used to determine the main interindividual plasma metabolome variation sources. In addition to the multivariate statistical method, Student’s t test was applied to analyze the significance of each metabolite. Logistic regression and receiver operating characteristic (ROC) analyses were used to evaluate the diagnostic value for MG. Statistical analyses were performed using SPSS software version 19.0 (IBM Corp., New York). All feature selection, logistic-regression model fitting, model coefficients, and cutoff determination were performed exclusively in the discovery set and subsequently applied unchanged to the validation set. For MS/MS fragments under standard collision-induced dissociation (CID) conditions can determine the total acyl chain length and total unsaturation, but cannot determine the specific position of the double bond, therefore, In subsequent analysis, we ignored information regarding the positions of double bonds in lipid compounds.

## Results

### Study design and baseline patient characteristics

One hundred and thirty participants were included in the discovery and validation sets. The discovery set included 40 participants, 20 of which had MG and 20 sex- and age-matched healthy controls. The validation set included 90 participants, 45 of which had MG and 45 sex- and age-matched healthy controls. Patients with MG were tested for the presence of anti-AChR antibodies. Except for plasma APOAI levels in the discovery set and Cr levels in the validation set, there were no significant differences in clinical biochemical parameters between the patients with MG and matched healthy controls. Among the 20 patients with MG in the discovery set, 15 were anti-AChR antibody positive, whereas 30 of the 45 patients with MG in the validation set were anti-AChR antibody positive. The baseline characteristics of the participants are shown in **Table 1**, and the workflow of the study is presented in **Figure 1**.

**Table 1:** Baseline characteristics of the patients in the discovery and validation sets.

Characteristics	Discovery set			Validation set		
	MG	Healthy controls	*P* value	MG	Healthy controls	*P* value
n	20	20	–	45	45	–
Men, n,%	9, 45%	9, 45%	–	20, 44.44%	21, 46.67%	0.832
Age, years	60.30±11.99	59.60±8.46	0.832	60.80±13.40	61.31±13.04	0.855
BW, Kg	67.65±7.57	64.95±11.85	0.396	68.63±12.62	67.71±12.93	0.751
HR, times/min	79.9±12.1	78.2±8.61	0.612	81.19±12.63	76.4±10.09	0.061
SBP, mm Hg	131.3±22.61	132.5±15.45	0.846	135.64±17.2	132.71±17.04	0.446
DBP, mm Hg	78.6±11.5	82.8±11.12	0.248	78.5±10.37	81.87±10.9	0.162
ALT, U/L	50.17±68.68	33.57±32.3	0.356	23.78±18.45	26.03±21.76	0.625
AST, U/L	23.94±18.46	24.52±19.32	0.924	22.34±11.2	22.91±10.12	0.813
TP, g/L	66.54±7.02	65.79±5.44	0.721	68.7±6.77	68.26±6.7	0.775
ALB, g/L	39.21±4.78	40.98±3.94	0.224	40.58±5.02	41.27±3.92	0.492
K, mmol/L	3.7±0.32	3.81±0.36	0.296	3.64±0.38	3.8±0.38	0.079
Na, mmol/L	141.01±4.1	140.24±2	0.455	140.64±2.78	140.49±2.39	0.792
Cl, mmol/L	102.79±2.63	103.47±2.97	0.445	101.21±17.64	105.51±3.57	0.118
Ca, mmol/L	2.18±0.15	2.24±0.11	0.159	2.24±0.12	2.28±0.12	0.144
P, mmol/L	0.97±0.28	1.09±0.21	0.124	1.08±0.2	1.06±0.23	0.773
Mg, mmol/L	0.82±0.09	0.85±0.07	0.224	0.85±0.14	0.87±0.1	0.475
Urea, mmol/L	5.76±3.02	5.33±1.44	0.594	5.11±1.02	5.67±2.17	0.161
Cr, μmol/L	62.49±15.75	64.33±11.16	0.682	59.03±12.02	69.68±19.76	0.006
UA, μmol/L	322±108.96	314.72±90.11	0.825	327.14±94.41	328.49±113.35	0.955
CysC, mg/L	0.99±0.16	0.9±0.19	0.118	0.97±0.29	0.89±0.2	0.164
CK, U/L	95.94±124.5	97±115.87	0.978	97.93±102.64	103.91±78.6	0.607
CK-MB, U/L	17.97±21.66	12.48±8.17	0.351	14.75±6.56	19.81±34.35	0.437
THCY, μmol/L	13.32±4.88	14.84±8.77	0.535	14.49±6.18	17.33±9.32	0.14
Lp(a), g/L	0.22±0.21	0.21±0.2	0.974	0.19±0.23	0.16±0.17	0.532
APOAI, g/L	0.98±0.21	1.12±0.17	0.046	1.15±0.2	1.09±0.11	0.172
APOB100, g/L	0.79±0.19	0.9±0.25	0.175	1.04±0.29	0.95±0.33	0.304
GLU, mmol/L	6.09±2.51	6.28±2.92	0.833	5.77±1.99	6.81±2.99	0.09
TCh, mmol/L	4.44±1.22	4.58±0.84	0.666	4.76±1.31	4.47±1.26	0.349
TG, mmol/L	1.6±0.97	1.56±0.95	0.888	1.71±0.91	1.62±0.8	0.662
LDL-C, mmol/L	2.54±0.85	2.6±0.72	0.817	3.04±0.95	2.53±1.02	0.065
AChR antibody positive, %	15,75%	–	–	30, 66.67%	–	–
General MG	18,90%	–	–	38, 84.44%	–	–
Ocular MG	2,10%	–	–	7, 15.56%	–	–
QMGS	8.05±6.11	–	–	8.56±5.17	–	–
MG-ADL	5.95±4.57	–	–	5.93±3.52	–	–
MGFA, n	Type IIIb:1; Type IIb:3; Type IIa:3; Type I:11; Type V:2			Type IIa:12; Type IIb:5; Type IIIa:1; Type IIIb:7; Type IVa:1; Type I:18; Type V:1		

BW, Body weight; HR, Heart rate; SBP, systolic blood pressure; DBP, diastolic blood pressure; ALT, Alanine aminotransferase; AST,Aspartate aminotransferase; TP, Total Protein; ALB, Albumin; K, potassium; Na, sodium; Cl, Chloride; Ca, calcium; P, Phosphonium; Mg, magnesium; Urea;Cr, creatinine; UA, uric acid; CysC, Cystatin C; CK, creatine Kinase; CK-MB, creatine kinase isoenzymes; THCY, Homocysteine; Lp(a), Lipoprotein A; APOA1, Apolipoprotein A1; APOB100, Apolipoprotein B100; GLU, glucose; TCh, Total Cholesterol; TG, triglyceride; LDL-C, low-Density Lipoprotein Cholesterol; AChR, anti-acetylcholine receptor; QMGS, Quantitative Myasthenia Gravis Score; MG-ADL, Myasthenia Gravis Activities of Daily Living; MGFA, Myasthenia Gravis Foundation of America.

**Figure 1:**
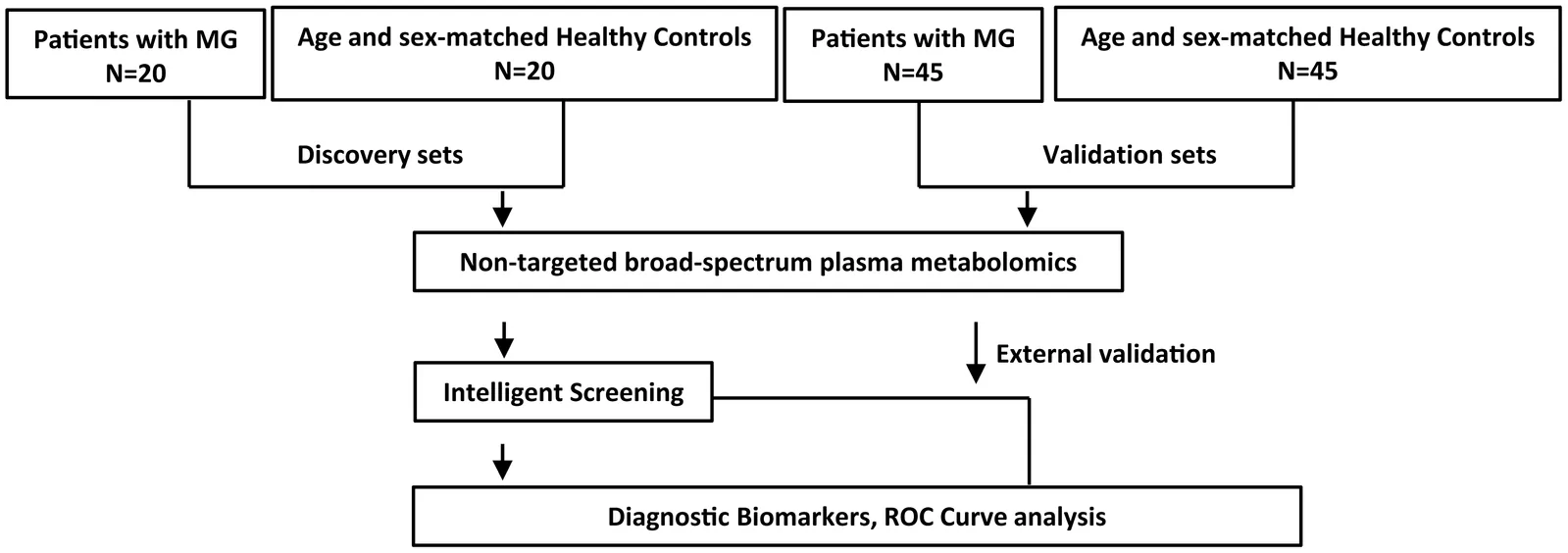
Study design.

### Plasma metabolic profile of patients with MG

To elucidate the metabolic profiles, a nontargeted plasma metabolomic method based on liquid chromatography–tandem mass spectrometry was used in positive and negative ion modes. During the analysis, the samples were arranged and processed randomly; the acquisition sequences of the samples in the discovery and validation sets are given in **Supplementary Tables S1, S2**.

The proportion of each metabolic feature across the quality control (QC) samples with a coefficient of variation (CV) <30% was 76.24%. An overlay of the QC total ion chromatograms in the discovery set is displayed in **Supplementary Figure S2**. Thus, these results indicated that the instrument exhibited good stability.

A total of 3212 and 3424 metabolites were identified in the discovery and validation sets, respectively. Additionally, a |log2(FoldChange) | value > 0.58 and *q* value <0.05 were set as the thresholds for screening differentially abundant metabolites. The results revealed 228 significantly increased metabolites and 98 significantly decreased metabolites in plasma from patients with MG relative to healthy controls in the discovery set, whereas 189 metabolites were increased and 209 were decreased the validation set (**Figures 2A, B**; **Supplementary Tables S3**, **S4**). The PCA model revealed clear separation between the patients with MG and healthy controls in both the discovery and validation sets evaluated in this study, indicating that MG is associated with profound systemic metabolic alterations (**Figure 2C**); PCA plots showing the clustering of the QC samples clustering are shown in **Supplementary Figure S3**. A Venn diagram revealed that 890 metabolites were detected in both the discovery and validation sets (**Figure 2D**; **Supplementary Table S5**). Metabolite set enrichment analysis revealed that steroid hormone biosynthesis was significantly enriched in discovery and validation sets, suggesting that alterations in steroid hormone metabolism may serve as potential pathological hallmarks of MG. Besides, pyrimidine and Pyrimidine metabolism, glycosaminoglycan biosynthesis–chondroitin sulfate/dermatan, sulfate and mannose type O-glycan biosynthesis were also significantly enriched either in the discovery or validation set (**Supplementary Table S6**). Further analysis revealed 41 metabolites with VIP values >1 among the 326 differentially abundant metabolites in the discovery sets (**Figure 2E**).

**Figure 2:**
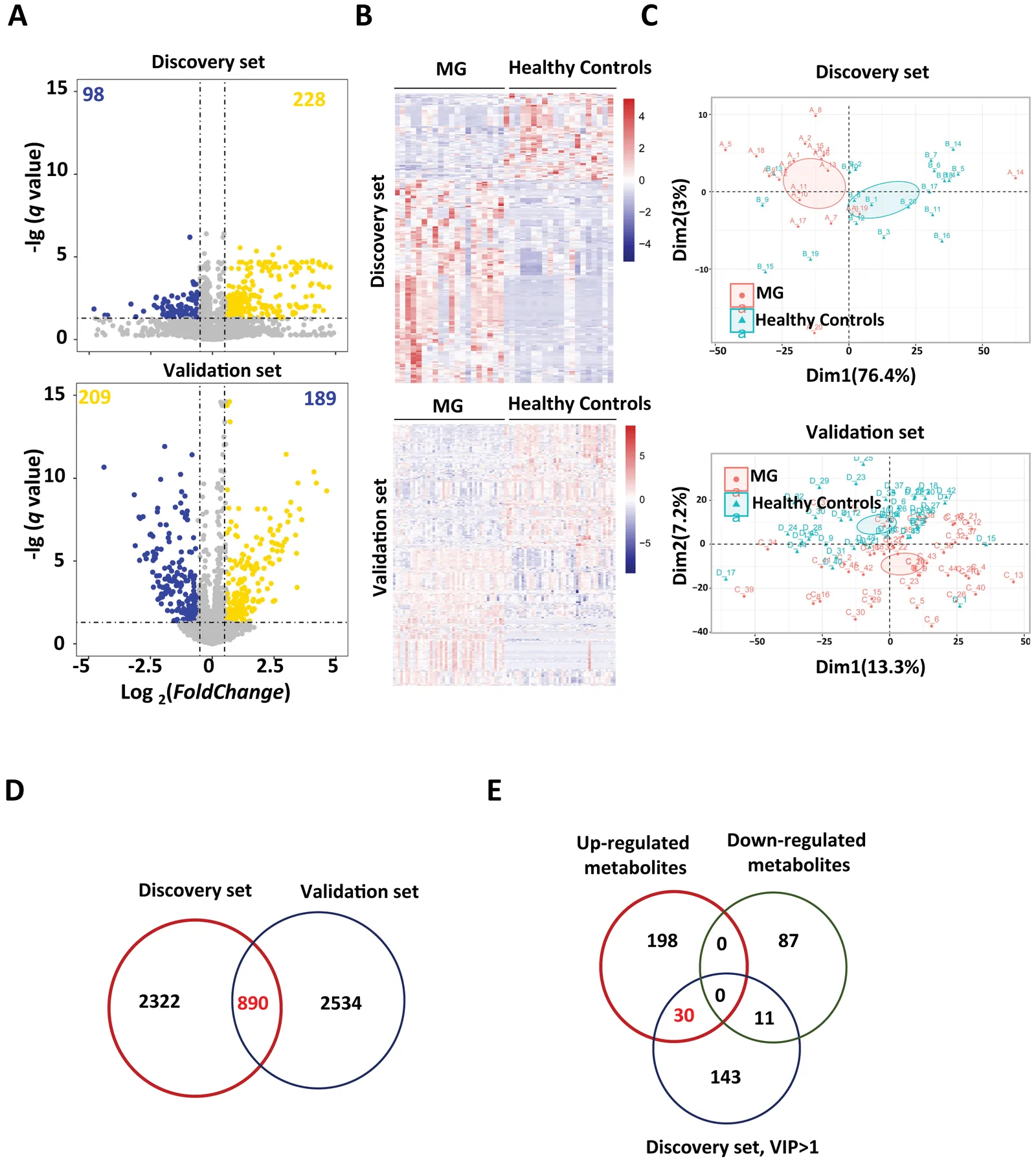
Characterization of the plasma metabolomic profile of patients with MG in the discovery and validation sets. **(A)** Volcano plots and **(B)** heatmaps of differentially abundant metabolites between patients with MG and healthy controls in the discovery and validation sets. **(C)** Principal component analysis (PCA) score plots of the metabolomic profiles between patients with MG and healthy controls in the discovery and validation sets. **(D)** Venn diagram illustrating metabolites that were detected in both the discovery and validation sets. **(E)** Venn diagram showing 41 metabolites with variable importance in projection (VIP) values of > 1 among the differential metabolites in discovery set.

### Plasma biomarker screening

Among these 41 metabolites, 15 were defined as level 1 on the basis of their compound level (**Figure 3A**). ROC curve analysis revealed that these 15 metabolites displayed significant diagnostic ability for MG in the discovery set (**Supplementary Table S7**). In our study, the metabolites with an area under the curve (AUC) of >0.8 and a F1_max_ score of >0.8 according to precision–recall (P-R) curve analyses were considered potential biomarkers for MG diagnosis. Finally, PE(O-18:1/0:0), PE(P-16:0/0:0) and LysoPE(P-18:1/0:0) were identified as potential metabolic biomarkers (**Figure 3B**; **Supplementary Table S7**). Among 65 MG patients, Pearson’s correlation analysis revealed no correlation between the plasma levels of the biomarkers and MG scores (**Supplementary Figure S4**).

**Figure 3:**
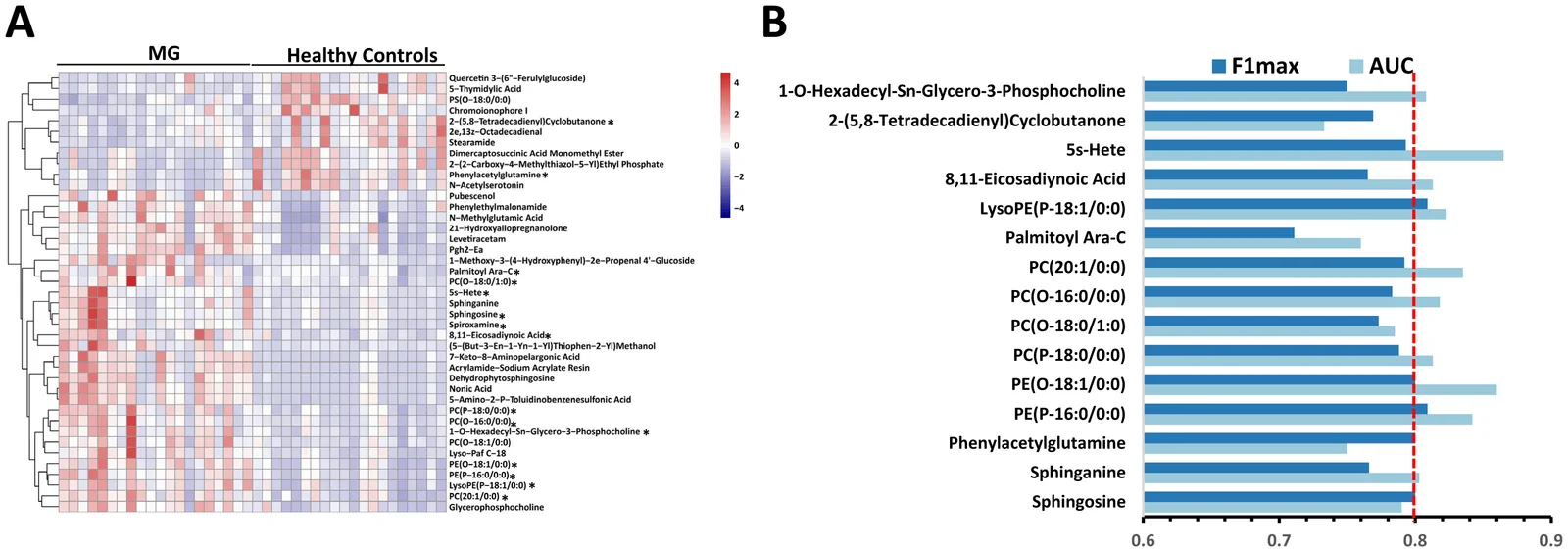
Plasma biomarker screening. **(A)** Heatmaps of 41 metabolites between patients with MG and healthy controls in the discovery set, 15 metabolites marked with * were defined as class 1 on the basis of their compound scores. **(B)** The metabolites with an area under the curve (AUC) of >0.8 and a F1_max_ score of >0.8 according to precision–recall (P-R) curve analyses were considered potential biomarkers for MG diagnosis.

### Validation of the diagnostic performance of the biomarker panel for MG

Among these 3 selected metabolites in the discovery set, plasma PE(O-18:1/0:0), PE(P-16:0/0:0) and LysoPE(P-18:1/0:0) levels were significantly higher in patients with MG than in healthy controls (**Figure 4A**). An ROC curve was used to assess the performance of these metabolic biomarkers for MG diagnosis, and the overall diagnostic performance of the biomarker panel for MG was determined using a logistic regression model. The results for the discovery set revealed that the MG diagnostic accuracy of the biomarker panel was better than that of the signaling metabolites. The AUC, sensitivity, and specificity of the plasma anti-AChR antibody test were 0.825, 65% and 100%, respectively, for the MG patients vs. healthy controls. Moreover, the screened biomarker panel exhibited significant diagnostic potential, with AUC, sensitivity, and specificity values for the plasma biomarker panel of 0.883, 75% and 90%, respectively, for patients with MG vs. healthy controls. When the biomarker panel was combined with the antibody assay results, the AUC, sensitivity, and specificity were 0.915, 80% and 95%, respectively, for the MG vs. healthy controls groups (**Figure 4B**; **Table 2**).

**Figure 4:**
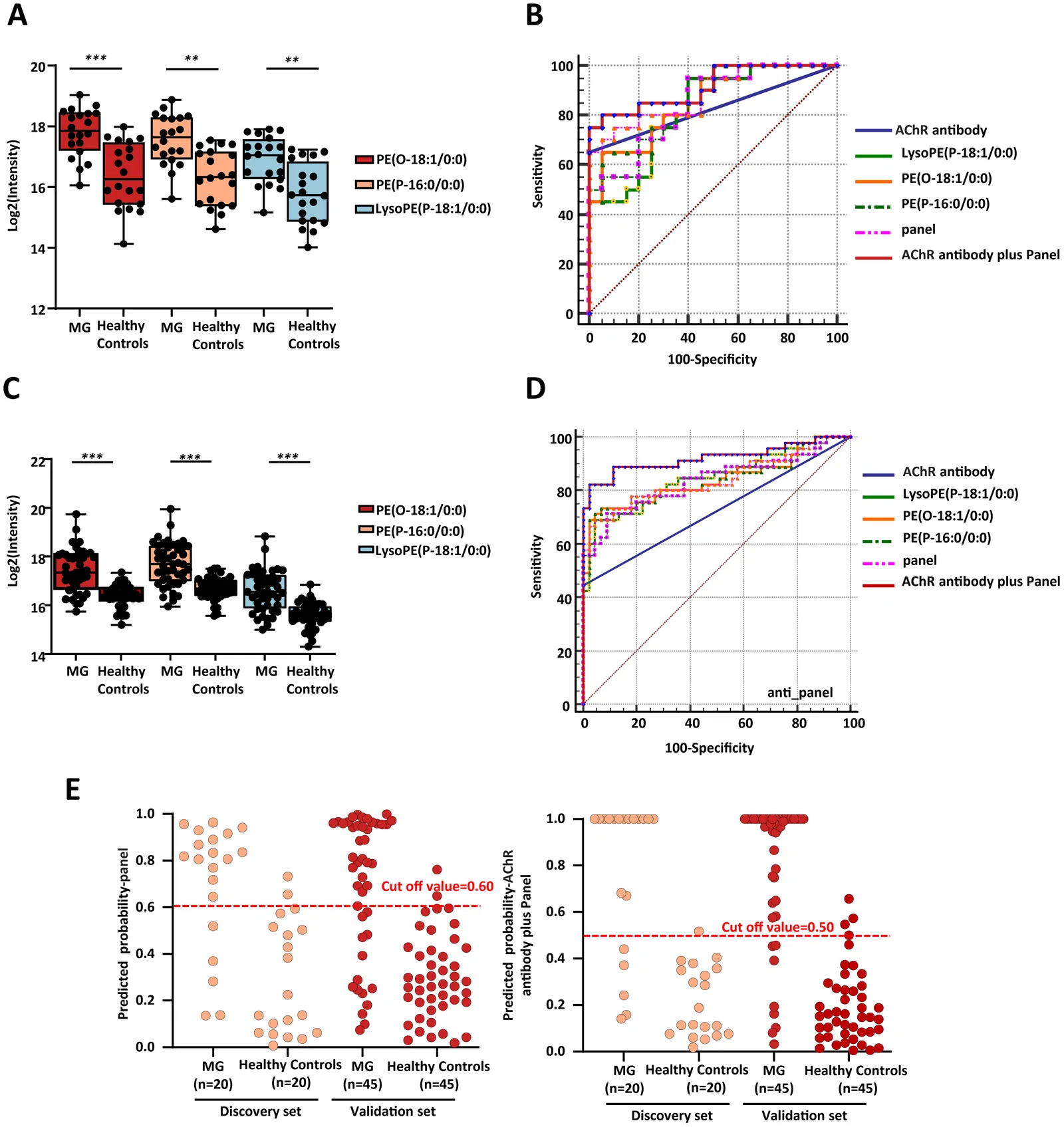
Validation of the MG diagnostic performance of the biomarker panel. **(A)** Box plots illustrating the differences in the plasma levels of the biomarkers in the panel between patients with MG and healthy controls in the discovery set. **(B)** The diagnostic outcomes in the discovery set are shown as ROC curves for comparisons between anti-AChR antibody testing, the biomarker panel, and the biomarker panel combined with anti-AChR antibody testing. **(C)** Box plots illustrating the differences in the plasma levels of the biomarkers in the panel between patients with MG and healthy controls in the validation set. **(D)** The diagnostic outcomes in the validation set are shown as ROC curves for comparisons between anti-AChR antibody testing, the biomarker panel, and the biomarker panel combined with anti-AChR antibody testing. **(E)** Comparison of the prediction accuracy of the biomarkers in the validation set between the biomarker panel and the biomarker panel plus anti-AChR antibody testing. Student *t* test, ^**^P < 0.01, and ^***^P < 0.001.

**Table 2:** MG diagnostic performance of the biomarkers.

Biomarker	Discovery set					Validation set				
	AUC	95% CI	*P* value	Sensitivity	Specificity	AUC	95% CI	*P* value	Sensitivity	Specificity
Traditional diagnosis										
AchR antibody testing	0.825	0.672-0.927	<0.0001	65	100	0.722	0.618-0.811	<0.0001	44	100
Newly screened biomarkers										
PE(O-18:1/0:0)	0.86	0.714-0.949	<0.0001	65	95	0.839	0.747-0.908	<0.0001	71	95
PE(P-16:0/0:0)	0.842	0.639-0.938	<0.0001	95	60	0.835	0.742-0.905	<0.0001	71	91
LysoPE(P-18:1/0:0)	0.823	0.669-0.925	<0.0001	95	60	0.845	0.753-0.913	<0.0001	68	95
Biomarker panel	0.883	0.741-0.962	<0.0001	75	90	0.843	0.752-0.911	<0.0001	68.89	95.56
AchR antibody testing plus Biomarker panel	0.915	0.783-0.980	<0.0001	80	95	0.921	0.845-0.968	<0.0001	82.22	97.78

Consistent trends were detected for PE(O-18:1/0:0), PE(P-16:0/0:0) and LysoPE(P-18:1/0:0) in plasma samples from patients with MG and healthy controls in the validation set (**Figure 4C**). The plasma anti-AChR antibody test was used to distinguish between patients with MG and healthy controls, with an AUC of 0.722, sensitivity of 44%, and specificity of 100%. Additionally, the AUC, sensitivity, and specificity of the plasma biomarker panel were 0.843, 68.89% and 95.56%, respectively, for the patients with MG vs. the healthy controls. Further analysis revealed that the combination of the biomarker panel and antibody assays improved the AUC to 0.921 (sensitivity of 82.22% and specificity of 97.78%) (**Figure 4D**; **Table 2**). In summary, in both the discovery and validation sets, compared to the antibody tests, the screened biomarkers demonstrated an equal ability to distinguish patients with MG from healthy controls; moreover, the combination of the biomarker panel and antibody testing significantly improved diagnostic accuracy.

In terms of the biomarker panel, on the basis of the greatest prediction sensitivity and specificity of the ROC curve in the discovery phase, the optimal cutoff value was determined to be 0.60 for patients with MG vs. healthy controls. We then used this cutoff value to predict which patients had MG in the validation set, and the predictive value was 80%. For the biomarker panel plus anti-AChR antibody testing, the optimal cutoff value in the discovery set and the predictive value in validation set were 0.5 and 100%, respectively (**Figure 4E**).

## Discussion

In our study, a metabolomics approach was used to evaluate the plasma metabolic profile of patients with MG. The results illustrated that altered metabolism may serve as a potential pathological hallmark of MG. A total of 3 plasma metabolites [PE(O-18:1/0:0), PE(P-16:0/0:0) and LysoPE(P-18:1/0:0)] were combined in a biomarker panel to differentiate patients with MG from healthy controls. Moreover, compared with antibody tests, the screened biomarkers demonstrated an equal ability to distinguish patients with MG from healthy controls; moreover, the combination of the biomarker panel and antibody assays significantly improved accuracy. However, further evaluation in independent cohorts including patients with suspected MG and other relevant autoimmune and neuromuscular junction disease controls is warranted.

In previous studies, altered profiles of plasma metabolites, including metal ions (20), cholesterol (21), and amino acids (22), have been reported; however, the detailed molecular mechanisms remain unclear, although the following reasons have been proposed (1). Changes in plasma metabolites may be attributable to alterations in the gut microbiota. An increasing number of studies have shown that the gut microbiota and fecal metabolites are not only closely associated with MG initiation but also influence MG subtype (23, 24). Additional studies have shown that oral probiotics can provide health benefits in several experimental models of autoimmune diseases and that the interaction between the gut microbiota and immune regulation must involve plasma metabolic pathways (2, 25). Mitochondrial dysfunction is involved in the initiation of MG through the impairment of muscle function and the proliferation and differentiation of immune cells (26). As energy factories, mitochondria are the primary energy suppliers for all life process and provide energy, especially for the heart, muscle and brain, which have high energy demands, via fatty acid beta-oxidation. Mitochondrial dysfunction leads to a decrease in fatty acid degradation, and abnormal plasma fatty acid levels are critical inducers of changes in the metabolic profile and immune responses (27). Consistent with these findings, the deficiency of lipin 1, a phosphatidic acid phosphohydrolase that regulates lipid metabolism, led to the development of MG in humans and zebrafish; moreover, lipin 1 plays a critical role in metabolic homeostasis (28, 29). In fact, dietary habits and the living environment significantly affect plasma metabolic profiles, although additional clinical data from large cohorts of patients with MG from multiple centers are needed to validate the relationship between metabolic profile alterations and MG.

Screening for specific MG biomarkers is necessary in clinical studies. Although plasma anti-AChR antibody positivity is considered a clinical diagnostic criterion, only 80% of patients with generalized MG and approximately 50% of patients with ocular MG are anti-AChR antibody positive, posing a significant obstacle to MG diagnosis (1, 30). In our study, the percentages of patients who were anti-AChR antibody positive were 75% and 66.67% in the discovery and validation sets, respectively, which are lower than those in the general patients with MG population. This discrepancy may be related to the inclusion of 10% of patients with ocular MG. An increasing number of studies have focused on the relationship between plasma metabolic profile alterations and MG, but there are currently no results that can be clinically translated because of issues such as sample size and study scale (31–33). In our study, we identified a plasma biomarker panel that included PE(O-18:1/0:0), PE(P-16:0/0:0) and LysoPE(P-18:1/0:0) that could be used to distinguish patients with MG from healthy controls and demonstrated good diagnostic performance in the discovery and validation sets.

In our study, MSEA of 890 metabolites revealed that steroid hormone biosynthesis pathway was significantly enriched in the discovery and validation sets. Steroid hormones like testosterone, progesterone, cortisol, aldosterone, and estradiol were considered as important endocrine chemical messengers that are involved in a vast number of physiological processes including metabolism, inflammation, immunity response, electrolyte and fluid balance, and secondary sex differentiation (34, 35). A significant amount of recent studies have illustrated that steroid hormone also was *de novo* biosynthesized in brain and peripheral nervous system tissues, except for the gonads, adrenal gland, placenta, intestines via a multi-step process that is catalyzed by members of the cytochrome P450 superfamily of monooxygenases and hydroxysteroid dehydrogenases (36, 37). Those physiological processes dysfunction was closely associated with MG onset under pathological condition. Peptide trophic hormones interacted with their cognate G protein-coupled receptors to initiate activation of steroidogenesis, while abnormal lipid metabolism have been shown to effect the steroidogenic pathway via regulating steroidogenic gene expression and activity as well as acting as secondary messengers in signaling cascades under the pathological conditions (38–40). Previous study showed that steroidogenesis was regulated by free polyunsaturated fatty acids (PUFA) and an increased PUFA content in phospholipids promoted steroidogenesis (41). A significant change in lipids metabolism was found in our study whether it’s the discovery or validation set, which was a key reason for the significant enrichment of the steroid hormone biosynthesis pathway. However, it remains unclear how the steroidogenesis leads to MG, and how the identified biomarkers regulate the onset of MG through the steroid hormone biosynthesis pathway. Except for steroid hormone biosynthesis pathway, the metabolic pathways enriched in the differentially abundant metabolites identified in our study, including fatty acid metabolism, amino acid metabolism, steroid hormone metabolism, and the citrate cycle (TCA cycle) either in the discovery or validation set, were reported in previous metabolomic studies on patients with MG (15, 42, 43). These findings demonstrated the reproducibility of the study results and highlight the important role of alterations in lipid metabolism in the pathological process of MG. However, whether in previous or our studies, since the pathway enrichment analyses were driven by relatively few metabolites, conclusions regarding pathway dysregulation should be interpreted cautiously.

With respect to the biomarker panel consisting of PE(O-18:1/0:0), PE(P-16:0/0:0) and LysoPE(P-18:1/0:0), although no reports have been published regarding the direct correlation between it and the pathological process of MG, abnormal plasma lipid levels were detected in a previous study. A study by Sikorski P. et al. revealed that plasma glycosphingolipid metabolism could reflect the pathological status of MG, as demonstrated by the increased abundance of phosphatidylethanolamines (PEs) in patients with MG (42). Indeed, PEs, as integral components of the plasma membrane, play a critical role in regulating immune cell function (44). A previous study revealed that an increase in PE levels or metabolism could promote immune cell differentiation and activation (45). However, significant increases in PE(O-18:1/0:0), PE(P-16:0/0:0) and LysoPE(P-18:1/0:0) levels in the plasma of patients with MG compared with those in healthy controls have not been reported in other studies, which may be attributable to discrepancies among different cohorts and analytical platforms.

This study has several limitations that warrant consideration. First, its single-center nature and small sample size reduce the statistical power and increase the risk of bias. Independent multicenter external validation is still needed before clinical implementation and to avoid overstating the immediate diagnostic utility of the biomarker panel. Furthermore, the results of our study revealed that the plasma levels of 3 metabolites were considered biomarkers with significant diagnostic performance for MG; however, the causal relationship between altered plasma biomarker levels and MG occurrence is not yet clear. Third, plasma metabolomic profiles are strongly influenced by fasting status, diet, body mass index, smoking status, alcohol consumption, physical activity, medication history, gut microbiota composition, and c circadian timing, etc. Although patients undergoing steroid and immunosuppressive treatments were excluded, additional information regarding fasting sample collection and other potential confounders was not included in our study because we did not have access to medical records, which may be among the factors affecting the accuracy of the results of this study. Besides, 65 healthy controls were recruited as controls in our study, it was difficult to discern whether these metabolic changes are specific to MG or represent a common signal across multiple autoimmune diseases. In the future, more appropriate controls (including patients with suspected MG and other relevant autoimmune and neuromuscular junction disorders) are needed to further validate the metabolic signatures of patients with MG. Finally, AChR-negative patients were included in our cohort, indeed, investigation of these patients was of great clinical utility. Nevertheless, it would be difficult to perform an exploratory analysis specifically among AChR-negative MG patients for a small sample size (5 of 20 patients with MG in the discovery set and 15 of 45 patients with MG in the validation set).

## Conclusions

In summary, the results of the present study revealed that the plasma metabolome of patients with MG was significantly different from that of the healthy controls in both the discovery and validation sets. Plasma PE(O-18:1/0:0), PE(P-16:0/0:0) and LysoPE(P-18:1/0:0) levels could be used as a biomarker panel to distinguish patients with MG from healthy controls across the two cohorts evaluated in this study; moreover, the metabolic biomarker panel had an equal diagnostic capability for MG, and compared with anti-AChR antibody testing alone, the combination of the biomarker panel and antibody assays significantly improved diagnostic accuracy. Differences in the plasma metabolomic profile may help to improve clinical MG diagnosis and elucidate the pathological mechanisms underlying MG. However, further evaluation in independent cohorts including patients with suspected MG and other relevant autoimmune and neuromuscular junction disease controls is warranted.

## Data Availability

The datasets presented in this study can be found in online repositories. The names of the repository/repositories and accession number(s) can be found in the article/**Supplementary Material**.

## References

[1] Gilhus NE Skeie GO Romi F Lazaridis K Zisimopoulou P Tzartos S . Myasthenia gravis - autoantibody characteristics and their implications for therapy. Nat Rev Neurol. (2016) 12:259–68. doi: 10.1038/nrneurol.2016.44 27103470

[2] Attarian S . New treatment strategies in myasthenia gravis. Rev Neurol (Paris). (2024) 180:971–81. doi: 10.1016/j.neurol.2024.09.006 39379218

[3] Meriggioli MN Sanders DB . Autoimmune myasthenia gravis: emerging clinical and biological heterogeneity. Lancet Neurol. (2009) 8:475–90. doi: 10.1016/S1474-4422(09)70063-8 19375665 PMC2730933

[4] Piehl F Eriksson-Dufva A Budzianowska A Feresiadou A Hansson W Hietala MA . Efficacy and safety of rituximab for new-onset generalized myasthenia gravis: the Rinomax randomized clinical trial. JAMA Neurol. (2022) 79:1105–12. doi: 10.1001/jamaneurol.2022.2887 36121672 PMC9486640

[5] Matsuki K Juji T Tokunaga K Takamizawa M Maeda H Soda M . Hla antigens in Japanese patients with myasthenia gravis. J Clin Invest. (1990) 86:392–9. doi: 10.1172/jci114724 1974553 PMC296740

[6] Matsuki K Maeda H Nomura Y Segawa M . Distortion of Hla gene transmission in childhood-onset myasthenia gravis. Lancet. (1992) 340:796. doi: 10.1016/0140-6736(92)92343-e 1356211

[7] Kerty E Elsais A Argov Z Evoli A Gilhus NE . Efns/Ens guidelines for the treatment of ocular myasthenia. Eur J Neurol. (2014) 21:687–93. doi: 10.1111/ene.12359 24471489

[8] Heldal AT Owe JF Gilhus NE Romi F . Seropositive myasthenia gravis: a nationwide epidemiologic study. Neurology. (2009) 73:150–1. doi: 10.1212/WNL.0b013e3181ad53c2 19597135

[9] Renton AE Pliner HA Provenzano C Evoli A Ricciardi R Nalls MA . A genome-wide association study of myasthenia gravis. JAMA Neurol. (2015) 72:396–404. doi: 10.1001/jamaneurol.2014.4103 25643325 PMC4856525

[10] Saruhan-Direskeneli G Hughes T Yilmaz V Durmus H Adler A Alahgholi-Hajibehzad M . Genetic heterogeneity within the Hla region in three distinct clinical subgroups of myasthenia gravis. Clin Immunol. (2016) 166-167:81–8. doi: 10.1016/j.clim.2016.05.003 27181991

[11] Narayanaswami P Sanders DB Wolfe G Benatar M Cea G Evoli A . International consensus guidance for management of myasthenia gravis: 2020 update. Neurology. (2021) 96:114–22. doi: 10.1212/wnl.0000000000011124 33144515 PMC7884987

[12] Mahic M Bozorg A DeCourcy J Golden K Gibson G Taylor C . Physician- and patient-reported perspectives on myasthenia gravis in Europe: a real-world survey. Orphanet J Rare Dis. (2023) 18:169. doi: 10.1186/s13023-023-02727-0 37386469 PMC10308674

[13] Petersson M Feresiadou A Jons D Ilinca A Lundin F Johansson R . Patient-reported symptom severity in a nationwide myasthenia gravis cohort: cross-sectional analysis of the Swedish Gemg study. Neurology. (2021) 97:e1382–91. doi: 10.1212/wnl.0000000000012604 34376512 PMC8520390

[14] Kaminski HJ Sikorski P Coronel SI Kusner LL . Myasthenia gravis: the future is here. J Clin Invest. (2024) 134(12):e179742. doi: 10.1172/JCI179742 39105625 PMC11178544

[15] Sha Q Zhang Z Li H Xu Y Wang J Du A . Serum metabolomic profile of myasthenia gravis and potential values as biomarkers in disease monitoring. Clin Chim Acta. (2024) 562:119873. doi: 10.1016/j.cca.2024.119873 39019424

[16] Punga AR Punga T . Circulating micrornas as potential biomarkers in myasthenia gravis patients. Ann N Y Acad Sci. (2018) 1412:33–40. doi: 10.1111/nyas.13510 29125182

[17] Beretta F Huang YF Punga AR . Towards personalized medicine in myasthenia gravis: role of circulating micrornas mir-30e-5p, mir-150-5p and mir-21-5p. Cells. (2022) 11(4):740. doi: 10.3390/cells11040740 35203389 PMC8870722

[18] Kang Y Li L Kang X Zhao Y Cai Y . Gut microbiota and metabolites in myasthenia gravis: early diagnostic biomarkers and therapeutic strategies. Clin Immunol. (2022) 245:109173. doi: 10.1016/j.clim.2022.109173 36351517

[19] Onishi Y Uzawa A Yasuda M Akamine H Ogaya E Handa H . Elevated serum levels of Il-10 family and Il-12 family cytokines in myasthenia gravis. J Neuroimmunol. (2025) 404:578621. doi: 10.1016/j.jneuroim.2025.578621 40288071

[20] Li K Hou L Tan Y Huang Y Shi J Han J . Iron metabolism patterns in non-anemic patients with myasthenia gravis: a cross-sectional and follow-up study. Front Neurol. (2022) 13:1060204. doi: 10.3389/fneur.2022.1060204 36504650 PMC9729274

[21] Paz ML Barrantes FJ . Cholesterol in myasthenia gravis. Arch Biochem Biophys. (2021) 701:108788. doi: 10.1016/j.abb.2021.108788 33548213

[22] Koslinski P Rzepinski L Koba M Gackowski M Maciejek Z . Amino acids levels as a potential biomarker in myasthenia gravis. Folia Neuropathol. (2022) 60:122–7. doi: 10.5114/fn.2022.114053 35359152

[23] Tan X Huang Y Chai T Zhao X Li Y Wu J . Differential gut microbiota and fecal metabolites related with the clinical subtypes of myasthenia gravis. Front Microbiol. (2020) 11:564579. doi: 10.3389/fmicb.2020.564579 33013794 PMC7506099

[24] Ding XJ Li HY Wang H Zhang XH Song M Jiang XH . Altered gut microbiota and metabolites in untreated myasthenia gravis patients. Front Neurol. (2023) 14:1248336. doi: 10.3389/fneur.2023.1248336 37789892 PMC10542407

[25] Rinaldi E Consonni A Guidesi E Elli M Mantegazza R Baggi F . Gut microbiota and probiotics: novel immune system modulators in myasthenia gravis? Ann N Y Acad Sci. (2018) 1413:49–58. doi: 10.1111/nyas.13567 29341125

[26] Chen J Lu J Lv Z Wang B Zhang S Xu P . Mitochondrial dysfunction in myasthenia gravis: exploring directions for future immunotherapy? A review. Biomol BioMed. (2025) 25:346–59. doi: 10.17305/bb.2024.11197 39388705 PMC11734830

[27] Wajner M Amaral AU . Mitochondrial dysfunction in fatty acid oxidation disorders: insights from human and animal studies. Biosci Rep. (2015) 36:e00281. doi: 10.1042/BSR20150240 26589966 PMC4718505

[28] Lu S Lyu Z Wang Z Kou Y Liu C Li S . Lipin 1 deficiency causes adult-onset myasthenia with motor neuron dysfunction in humans and neuromuscular junction defects in zebrafish. Theranostics. (2021) 11:2788–805. doi: 10.7150/thno.53330 33456573 PMC7806489

[29] Miranda M Escote X Alcaide MJ Solano E Ceperuelo-Mallafre V Hernandez P . Lpin1 in human visceral and subcutaneous adipose tissue: similar levels but different associations with lipogenic and lipolytic genes. Am J Physiol Endocrinol Metab. (2010) 299:E308–17. doi: 10.1152/ajpendo.00699.2009 20530740

[30] Vinciguerra C Bevilacqua L Lupica A Ginanneschi F Piscosquito G Rini N . Diagnosis and management of seronegative myasthenia gravis: lights and shadows. Brain Sci. (2023) 13(9):1286. doi: 10.3390/brainsci13091286 37759888 PMC10526522

[31] Gu S Wang X Yang H Wang Y Yan C Lin X . Identification of metabolic biomarkers and therapeutic targets in the thymoma-associated myasthenia gravis treated with methylprednisolone. Discov Oncol. (2025) 16:926. doi: 10.1007/s12672-025-02700-2 40418396 PMC12106182

[32] Billig HE Jr. Morehouse LE . Performance and metabolic alterations during betaine glycocyamine feeding in myasthenia gravis. Arch Phys Med Rehabil. (1955) 3636(4):233–6. 14362739

[33] Bhandage AK Kenina V Huang YF Roddate M Kauke G Grosmane A . Serum protein biomarker profile distinguishes acetylcholine receptor antibody seropositive myasthenia gravis patients from healthy controls. iScience. (2024) 27:110564. doi: 10.1016/j.isci.2024.110564 39165841 PMC11334828

[34] Lucki NC Sewer MB . Multiple roles for sphingolipids in steroid hormone biosynthesis. Sub-cell Biochem. (2008) 49:387–412. doi: 10.1007/978-1-4020-8831-5_15 18751920 PMC4104360

[35] Keith SA . Steroid hormone regulation of innate immunity in Drosophila melanogaster. PloS Genet. (2023) 19:e1010782. doi: 10.1371/journal.pgen.1010782 37319172 PMC10270360

[36] Ghayee HK Auchus RJ . Basic concepts and recent developments in human steroid hormone biosynthesis. Rev Endocr Metab Disord. (2007) 8:289–300. doi: 10.1007/s11154-007-9052-2 17926129

[37] Mellon SH . Neurosteroid regulation of central nervous system development. Pharmacol Ther. (2007) 116:107–24. doi: 10.1016/j.pharmthera.2007.04.011 17651807 PMC2386997

[38] Bollag WB . Role of phospholipases in adrenal steroidogenesis. J Endocrinol. (2016) 229:R29–41. doi: 10.1530/JOE-16-0007 26878860

[39] Miller WL . Mechanism of Star's regulation of mitochondrial cholesterol import. Mol Cell Endocrinol. (2007) 265-266:46–50. doi: 10.1016/j.mce.2006.12.002 17207924

[40] Urs AN Dammer E Kelly S Wang E Merrill AH Jr. Sewer MB . Steroidogenic factor-1 is a sphingolipid binding protein. Mol Cell Endocrinol. (2007) 265-266:174–8. doi: 10.1016/j.mce.2006.12.016 17196738 PMC1850975

[41] Aderhold A Alexaki VI . Lipid metabolism in the adrenal gland. Front Endocrinol (Lausanne). (2025) 16:1577505. doi: 10.3389/fendo.2025.1577505 40551885 PMC12183020

[42] Sikorski P Li Y Cheema M Wolfe GI Kusner LL Aban I . Serum metabolomics of treatment response in myasthenia gravis. PloS One. (2023) 18:e0287654. doi: 10.1371/journal.pone.0287654 37816000 PMC10564178

[43] Wei B Yuan H He J Hu Y Li J Wu X . Untargeted serum metabolomic profiling in patients with generalized and ocular myasthenia gravis. MC Ophthalmol. (2026) 26(1):323. doi: 10.1186/s12886-026-04843-4 42070048 PMC13251279

[44] Zhang T de Waard AA Wuhrer M Spaapen RM . The role of glycosphingolipids in immune cell functions. Front Immunol. (2019) 10:90. doi: 10.3389/fimmu.2019.00090 30761148 PMC6361815

[45] Aldrovandi M Hinz C Lauder SN Podmore H Hornshaw M Slatter DA . Dioxolanea3-phosphatidylethanolamines are generated by human platelets and stimulate neutrophil integrin expression. Redox Biol. (2017) 11:663–72. doi: 10.1016/j.redox.2017.01.001 28160743 PMC5288459

