# Supplementary material for: Comprehensive metabolomic characterization of patients with myasthenia gravis
Source: Front Immunol. 2026 Sep 29;17:1906581. doi: 10.3389/fimmu.2026.1906581 (PMC13657992; doi:10.3389/fimmu.2026.1906581)
Supplement: Supplementary file 1 [file DataSheet1.docx]

**Comprehensive Metabolomic Characterization of Patients with Myasthenia Gravis**

Hui Deng^1#^, Yuexuan Wang^2#^, Xiaowen Yang^3^, Jing Qin^4^, Jing Liu^3^, Tianlong Liu^3,5,6*^

1Department of Neurology, Affiliated Hospital of Inner Mongolia Medical University, Hohhot, China, 2College of Pharmacy, Inner Mongolia Medical University, Hohhot, China, 3 Department of Pharmacy, Affiliated Hospital of Inner Mongolia Medical University, Hohhot, China,4Department of Endocrinology, Affiliated Hospital of Inner Mongolia Medical University, Hohhot, China, 5Key Laboratory of Clinical and Basic Research on Cardiovascular Diseases, Basic Research Team of Cardiovascular Diseases, Affiliated Hospital of Inner Mongolia Medical University, Hohhot, China, 6Inner Mongolia Autonomous Region Institute of Cardiovascular Diseases, Hohhot, China

^#^ These authors contributed equally to this work.

^*^**Correspondence to:**

**Tianlong Liu, PhD,** Department of Pharmacy, Affiliated Hospital of Inner Mongolia Medical University, No. 1, Tongdao North Street, Huimin District, Hohhot 010059, China.

E-mail: tianlongliu1984@immu.edu.cn

Phone: Tel: +86-0471-3451657

**Running title:** Plasma Metabolomic Characterization of MG

**Figure S1 The statistical analysis workflow of non-targeted metabolomics data.**

**Figure S2 The overlap of QC total ion chromatograms under positive(A) and negative(B) ion mode in discovery set.**

**Figure S3 Principal component analysis (PCA) score plots of metabolomic profiles between patients with MG and healthy controls in discovery (A) and validation(B) sets.**

**Figure S4 The Pearson correlation coefficient between MG scores and plasma biomarker level.**

**Table S1 The acquisition sequence of sample in discovery set.**

| **sample** | **class** | **order** | **batch** |
| --- | --- | --- | --- |
| QC1 | QC | 6 | 1 |
| A1 | sample | 7 | 1 |
| A2 | sample | 8 | 1 |
| A3 | sample | 9 | 1 |
| A4 | sample | 10 | 1 |
| A5 | sample | 11 | 1 |
| A6 | sample | 12 | 1 |
| A7 | sample | 13 | 1 |
| A8 | sample | 14 | 1 |
| A9 | sample | 15 | 1 |
| A10 | sample | 16 | 1 |
| QC2 | QC | 17 | 1 |
| A11 | sample | 18 | 1 |
| A12 | sample | 19 | 1 |
| A13 | sample | 20 | 1 |
| A14 | sample | 21 | 1 |
| A15 | sample | 22 | 1 |
| A16 | sample | 23 | 1 |
| A17 | sample | 24 | 1 |
| A18 | sample | 25 | 1 |
| A19 | sample | 26 | 1 |
| A20 | sample | 27 | 1 |
| QC3 | QC | 28 | 1 |
| B1 | sample | 29 | 1 |
| B2 | sample | 30 | 1 |
| B3 | sample | 31 | 1 |
| B4 | sample | 32 | 1 |
| B5 | sample | 33 | 1 |
| B6 | sample | 34 | 1 |
| B7 | sample | 35 | 1 |
| B8 | sample | 36 | 1 |
| B9 | sample | 37 | 1 |
| B10 | sample | 38 | 1 |
| QC4 | QC | 39 | 1 |
| B11 | sample | 40 | 1 |
| B12 | sample | 41 | 1 |
| B13 | sample | 42 | 1 |
| B14 | sample | 43 | 1 |
| B15 | sample | 44 | 1 |
| B16 | sample | 45 | 1 |
| B17 | sample | 46 | 1 |
| B18 | sample | 47 | 1 |
| B19 | sample | 48 | 1 |
| B20 | sample | 49 | 1 |
| QC5 | QC | 50 | 1 |

**Table S2 The acquisition sequence of sample in validation set.**

| **sample** | **class** | **order** | **batch** |
| --- | --- | --- | --- |
| QC1 | QC | 6 | 1 |
| C1 | sample | 7 | 1 |
| C2 | sample | 8 | 1 |
| C3 | sample | 9 | 1 |
| C4 | sample | 10 | 1 |
| C5 | sample | 11 | 1 |
| C6 | sample | 12 | 1 |
| C7 | sample | 13 | 1 |
| C8 | sample | 14 | 1 |
| C9 | sample | 15 | 1 |
| QC2 | QC | 16 | 1 |
| C10 | sample | 17 | 1 |
| C11 | sample | 18 | 1 |
| C12 | sample | 19 | 1 |
| C13 | sample | 20 | 1 |
| C14 | sample | 21 | 1 |
| C15 | sample | 22 | 1 |
| C16 | sample | 23 | 1 |
| C17 | sample | 24 | 1 |
| C18 | sample | 25 | 1 |
| QC3 | QC | 26 | 1 |
| C19 | sample | 27 | 1 |
| C20 | sample | 28 | 1 |
| C21 | sample | 29 | 1 |
| C22 | sample | 30 | 1 |
| C23 | sample | 31 | 1 |
| C24 | sample | 32 | 1 |
| C25 | sample | 33 | 1 |
| C26 | sample | 34 | 1 |
| C27 | sample | 35 | 1 |
| QC4 | QC | 36 | 1 |
| C28 | sample | 37 | 1 |
| C29 | sample | 38 | 1 |
| C30 | sample | 39 | 1 |
| C31 | sample | 40 | 1 |
| C32 | sample | 41 | 1 |
| C33 | sample | 42 | 1 |
| C34 | sample | 43 | 1 |
| C35 | sample | 44 | 1 |
| C36 | sample | 45 | 1 |
| QC5 | QC | 46 | 1 |
| C37 | sample | 47 | 1 |
| C38 | sample | 48 | 1 |
| C39 | sample | 49 | 1 |
| C40 | sample | 50 | 1 |
| C41 | sample | 51 | 1 |
| C42 | sample | 52 | 1 |
| C43 | sample | 53 | 1 |
| C44 | sample | 54 | 1 |
| C45 | sample | 55 | 1 |
| QC6 | QC | 56 | 1 |
| D1 | sample | 57 | 1 |
| D2 | sample | 58 | 1 |
| D3 | sample | 59 | 1 |
| D4 | sample | 60 | 1 |
| D5 | sample | 61 | 1 |
| D6 | sample | 62 | 1 |
| D7 | sample | 63 | 1 |
| D8 | sample | 64 | 1 |
| D9 | sample | 65 | 1 |
| QC7 | QC | 66 | 1 |
| D10 | sample | 67 | 1 |
| D11 | sample | 68 | 1 |
| D12 | sample | 69 | 1 |
| D13 | sample | 70 | 1 |
| D14 | sample | 71 | 1 |
| D15 | sample | 72 | 1 |
| D16 | sample | 73 | 1 |
| D17 | sample | 74 | 1 |
| D18 | sample | 75 | 1 |
| QC8 | QC | 76 | 1 |
| D19 | sample | 77 | 1 |
| D20 | sample | 78 | 1 |
| D21 | sample | 79 | 1 |
| D22 | sample | 80 | 1 |
| D23 | sample | 81 | 1 |
| D24 | sample | 82 | 1 |
| D25 | sample | 83 | 1 |
| D26 | sample | 84 | 1 |
| D27 | sample | 85 | 1 |
| QC9 | QC | 86 | 1 |
| D28 | sample | 87 | 1 |
| D29 | sample | 88 | 1 |
| D30 | sample | 89 | 1 |
| D31 | sample | 90 | 1 |
| D32 | sample | 91 | 1 |
| D33 | sample | 92 | 1 |
| D34 | sample | 93 | 1 |
| D35 | sample | 94 | 1 |
| D36 | sample | 95 | 1 |
| QC10 | QC | 96 | 1 |
| D37 | sample | 97 | 1 |
| D38 | sample | 98 | 1 |
| D39 | sample | 99 | 1 |
| D40 | sample | 100 | 1 |
| D41 | sample | 101 | 1 |
| D42 | sample | 102 | 1 |
| D43 | sample | 103 | 1 |
| D44 | sample | 104 | 1 |
| D45 | sample | 105 | 1 |
| QC11 | QC | 106 | 1 |
| QC12 | QC | 107 | 1 |

**Table S6 Metabolite set enrichment analysis of 890 metabolites in discover and validation sets.**

| **MSEA (MG plus healthy controls in discovery set)** | | | | | | | | | |  |
| --- | --- | --- | --- | --- | --- | --- | --- | --- | --- | --- |
| **Terms** | **Total Cmpd** | | **Hits** | | **Statistic Q** | **Expected Q** | **Raw p** | **Holm p** | **FDR** | **Metabolites** |
| Phenylalanine metabolism | 8 | | 2 | | 23.395 | 2.561 | 9.9138E-5 | 0.004263 | 0.0022267 | Phenylalanine; Phenylethylamine |
| **Steroid hormone biosynthesis** | 86 | | 1 | | 33.08 | 2.5641 | 0.00010357 | 0.0043498 | 0.0022267 | Cortisone |
| Pyrimidine metabolism | 39 | | 2 | | 18.428 | 2.5641 | 0.00074488 | 0.03054 | 0.010677 | Orotic acid;Thymidine |
| **MSEA (MG plus healthy controls in validation set)** | | | | | | | | | |  |
| **Terms** | | **Total Cmpd** | | **Hits** | **Statistic Q** | **Expected Q** | **Raw p** | **Holm p** | **FDR** | **Metabolites** |
| Valine, leucine and isoleucine degradation | | 40 | | 1 | 53.324 | 1.1236 | 3.1704e-16 | 1.3633e-14 | 6.8165e-15 | Isoleucine |
| Valine, leucine and isoleucine biosynthesis | | 8 | | 1 | 53.324 | 1.1236 | 3.1704e-16 | 1.3633e-14 | 6.8165e-15 | Isoleucine |
| Taurine and hypotaurine metabolism | | 8 | | 3 | 29.256 | 1.1236 | 2.16e-09 | 8.8561e-08 | 3.096e-08 | Taurine; 5-L-Glutamyl-taurine; Taurocholic acid |
| Mannose type O-glycan biosynthesis | | 4 | | 1 | 20.772 | 1.1236 | 6.363e-06 | 0.00025452 | 5.4722e-05 | Serine |
| Glycosaminoglycan biosynthesis - chondroitin sulfate / dermatan sulfate | | 3 | | 1 | 20.772 | 1.1236 | 6.363e-06 | 0.00025452 | 5.4722e-05 | Serine |
| Sphingolipid metabolism | | 33 | | 3 | 11.901 | 1.1236 | 0.0001216 | 0.0046209 | 0.00087148 | Sphingosine 1-phosphate; Phytosphingosine;  Serine; |
| Primary bile acid biosynthesis | | 46 | | 6 | 12.188 | 1.1236 | 0.00021784 | 0.00806 | 0.0013381 | 7a-Hydroxy-cholestene-3-one; Chenodeoxycholic acid glycine conjugate; Taurine; 7alpha-Hydroxy-3-oxo-4-cholestenoate;  Glycocholic acid; Taurocholic acid |
| **Steroid hormone biosynthesis** | | 86 | | 1 | 9.6646 | 1.1236 | 0.0028608 | 0.10299 | 0.015377 | Cortisone |
| beta-Alanine metabolism | | 21 | | 1 | 7.7542 | 1.1236 | 0.00787 | 0.27545 | 0.037601 | Spermine |

**Table S7 Diagnostic performance of 15 metabolites for MG in discovery set.**

| **Metabolites** | **AUC** | **SE** | **95% CI** | **F1_max_** | **Results** |
| --- | --- | --- | --- | --- | --- |
| Sphingosine | 0.79 | 0.0726 | 0.632 to 0.902 | 0.8 |  |
| Sphinganine | 0.803 | 0.0695 | 0.646 to 0.911 | 0.766 |  |
| Phenylacetylglutamine | 0.75 | 0.0814 | 0.588 to 0.873 | 0.8 |  |
| **PE(P-16:0/0:0)** | **0.842** | **0.0606** | **0.693 to 0.938** | **0.809** | **🗸** |
| **PE(O-18:1(9Z)/0:0)** | **0.86** | **0.0566** | **0.714 to 0.949** | **0.8** | **🗸** |
| PC(P-18:0/0:0) | 0.813 | 0.0724 | 0.658 to 0.918 | 0.788 |  |
| PC(O-18:0/1:0) | 0.785 | 0.0764 | 0.627 to 0.899 | 0.773 |  |
| PC(O-16:0/0:0) | 0.818 | 0.0705 | 0.664 to 0.922 | 0.783 |  |
| PC(20:1(13Z)/0:0) | 0.835 | 0.0661 | 0.684 to 0.933 | 0.792 |  |
| Palmitoyl Ara-C | 0.76 | 0.0787 | 0.599 to 0.881 | 0.711 |  |
| **LysoPE(P-18:1(9Z)/0:0)** | **0.823** | **0.0652** | **0.669 to 0.925** | **0.809** | **🗸** |
| 8,11-Eicosadiynoic Acid | 0.813 | 0.0707 | 0.658 to 0.918 | 0.765 |  |
| 5s-Hete | 0.865 | 0.0557 | 0.720 to 0.952 | 0.793 |  |
| 2-(5,8-Tetradecadienyl)Cyclobutanone | 0.733 | 0.0796 | 0.569 to 0.860 | 0.769 |  |
| 1-O-Hexadecyl-Sn-Glycero-3-Phosphocholine | 0.808 | 0.0687 | 0.652 to 0.915 | 0.75 |  |
